# The skills required for transition to university and study in biological sciences: A student perspective

**DOI:** 10.1002/2211-5463.70234

**Published:** 2026-03-31

**Authors:** Janella Borrell, Susan Crennell

**Affiliations:** ^1^ Department of Life Sciences University of Bath UK

**Keywords:** curriculum review, skills development, transition to university

## Abstract

In preparation for a university‐wide Curriculum Transformation project across the University of Bath, a survey of student opinions of the skills training they received, required and developed during their bioscience studies was undertaken. Separate focus group discussions with first and final‐year students defined the parameters of initial and follow‐up questionnaires used to determine perceived confidence in 16 different transferrable skills. With responses to the first questionnaire from 204 students (23% of the cohort) the sample size was sufficient to perform statistical analysis on the results. The responses to both questionnaires showed, that by the end of their course the students valued having improved their academic/transferrable skills more than their practical laboratory skills. The survey data and focus group discussions revealed that teamwork, study, time management and organisational skills were perceived to be well developed before arrival at university. While some skills were recognised as having improved through formal teaching (Academic and scientific writing, research, laboratory, analytical and presentation skills), other more transferrable skills were thought to have improved as they are inherent to successful independent living at university and on placement. Actionable suggestions were made for enhancements to formal teaching of important transferrable skills that are not addressed in the current provision such as leadership (through role rotation in group work), resilience and problem‐solving (e.g. in practical classes requiring adjustment to succeed). This work suggests that many important transferrable skills are acquired inherently, allowing formal skills training to focus on communication, laboratory, research, leadership, resilience and problem‐solving.

## Graphical abstract

Bioscience students were asked for their opinions on the value and teaching of skills. 204 responded that teamwork, time management and study skills are necessary to reach University, that scientific writing, research, laboratory and presentation skills are taught effectively during their studies, while other skills are gained inherently through study in a new environment. Transferrable skills become increasingly important.

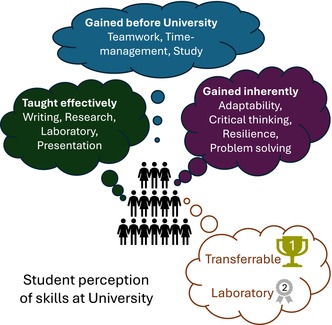

**Abbreviations**
ANOVA: analysis of variance
COVID: corona virus disease
GDPR: general data protection regulation (EU)
JISC‐AGCAS: Joint Information Systems Committee‐ Association of Graduate Careers Advisory Services partnership
SPSS: statistical package for social sciences
UK: United Kingdom

Inherent to a science degree is the learning of subject‐specific and transferrable skills, yet the skills chosen by academics to teach may not align with those perceived as important by students. This study was undertaken to identify the skills that bioscience students think require development during their study and to understand their view on which require formal training rather than being inherent to university life. This work was undertaken in the context of curriculum redesign at the University of Bath, with the opportunity to inform the content of new skills units in bioscience degrees.

The purpose of a university education has been the subject of debate for centuries. Newman's idea of a ‘university man,’ (1853), included cognitive skills (for example, clarity of thought and analysis), communication and interpersonal skills, as well as certain affective qualities. He believed that higher education, as well as fulfilling the individual, also improved the society [1].

Following the drive to regulate public expenditure in the early 80s, the government and employers challenged the classical autonomy of the university sector [2] leading to a crisis in the role of higher education in the late 90s with arguments for and against employability being a core purpose of higher education still ongoing [3]. This challenge led to an increasing focus on ‘personal transferable skills’ and ‘generic competences’ as a way to enhance the value of degrees [4]. There was also a ‘lessening of concern about what a graduate needs to *know* and an increasing interest in what she or he needs to be able to *do*.’ [5].

Presently, amid the rising global economic uncertainty, employers are seeking graduates with an array of transferable skills that promote flexibility and adaptability in the workplace. These are essential for navigating through a successful career in this dynamic world, where employees are likely to encounter unexpected situations and adapt appropriately [6].

Additionally, the rise in mass higher education has led to an increasingly competitive graduate labour market [7]. As a result, students are recognising that their academic credentials are no longer sufficient to shape their employment outcomes and are beginning to appreciate the importance of developing ‘soft credentials,’ which are not specific to their subject [8]. Bioscience students undertake a wide range of employment opportunities; the JISC AGCAS report (2023) shows only 23% of UK Biology graduates are working as science professionals 15 months following graduation, so the majority will be drawing primarily on these transferrable skills for their employment [9]. These transferable skills are also important for aiding formal university study, especially during the first year of university as students adapt to new styles of teaching and learning [10].

A successful transition from school to university continues to be a considerable challenge for many first‐year students [11]. Consequently, the majority of university withdrawals worldwide occur within or after the first year of university [12]. It is undeniable that the leap from school to higher education is extensive, especially for biosciences courses where students are traditionally taught in large, anonymous lecture theatres for the first time [13]. It is often argued that students enter university with minimal experience of self‐directed learning as, at school in the UK, pupils are equipped only with the information necessary to pass exams [14]. This is confirmed by a longitudinal study looking at the self‐assessment of 31 Key skills (as defined by the Assessment and Qualification Alliance) of first‐year medical undergraduates [15] where there was a significant decrease in laboratory, data handling and numeracy skills following Curriculum 2000 changes to post‐16 education. As a result, students must quickly develop these technical skills and also vital life skills such as time management and resilience to succeed at university level.

Previous work by Scott [16] and Demaria, Hodgson and Czech [17] has revealed that by introducing a compulsory skills unit into the university curriculum, students' confidence in performing skills‐related tasks increases accordingly, alongside their awareness and understanding of skills development. Such a skills unit has been part of the biosciences curriculum at the University of Bath for some time, focussing primarily on practical skills development, but also incorporating mathematical training and communication (presentation and essay‐writing) skills, the last in a small group tutorial context. In this study we took the opportunity of a university‐wide curriculum redesign to investigate the student view of the skills that should be taught in the first year of biosciences degrees.

Although various studies have been carried out on skills development in higher education, many of them concentrated on only one specific skill, for example: academic writing [18], research [19] or time management [20], while some explored two or three skills [17]. However, few studies address a wider range of skills in this context, and those that do e.g. [15] either have not designed their study around the skills students perceive as important or not examined this in the context of their existing abilities. Timming *et al*. (2025) looked at the difference in perception of the importance of 24 graduate attributes between job seekers and managers, however the skills examined were chosen through literature search rather than from student perceptions [21]. The valuable work by Scott [16] looks at bioscience student confidence in their skills acquisition rather than whether the skills taught are those the students most value. Thus, we undertook this study with the aims of understanding which skills are valued by students at university and also how they perceive they attain these skills. Knowing both which skills are valued by students and which of these require formal tuition is crucial for the design of science curricula where skills tuition time is necessarily limited. Although the work was carried out in our local context, the questions were not tied to particular details of taught units so are likely to be similar to views held by Biosciences students more broadly.

In the first year of their Bioscience study at the University of Bath, the students undertake a skills module combining practical classes with training in essay and report writing, and oral presentations; these last in a small group tutorial setting. Through focus groups and questionnaires aimed at all years of Bioscience and Natural Sciences students at the University of Bath we asked students for their opinions on the skills they had acquired and the benefits of the training they received, and evaluated how these changed as they progressed through the years of study. This work was initiated to evaluate the effectiveness and appropriateness of the content of the existing skills module and inform the redevelopment of Skills teaching in both bioscience and interdisciplinary Natural Sciences degrees at the University of Bath as part of the university‐wide Curriculum Transformation project. Our hypotheses were that all skills required some measure of formal teaching, and that a skills module was required for the students to gain and recognise that they had gained these skills. Our results showed that students perceive several important skills, such as teamwork and organisation, are gained before arrival and honed inherently through being at university. In consequence, less emphasis can be placed on these in the curriculum. This finding is further validated by skills that of necessity have to be taught at university (such as academic and scientific writing, presentation, laboratory and research skills) being recognised as having been acquired through formal training. The training in laboratory skills is the most overt, being in a unique environment, and this was the skill in which the students were most satisfied with their training. Leadership, resilience and problem‐solving were skills the students considered could have been included in their skills training to improve their degree. Identifying which skills require formal teaching will enable the focus of necessarily limited resources on these, improving the skills that students perceive as important. This work should be of interest to others reflecting on their practice in this area or embarking on course redesign.

## Methods

### Ethics

Both questionnaires used in this study were anonymous and did not collect identifying information from participants. The focus group participants volunteered, each signed a consent form explaining that their personal data would not be stored and views would be confidential and anonymised (File S1). This informed consent procedure was granted ethical approval by the University of Bath Social Sciences Research Ethics Committee (ethics application 6273–7685). The study methodology conforms to the standards set by the Declaration of Helsinki.

### Focus groups were used to define the skills to be included

The first stage was to define the skills to be investigated. The parameters of the study were defined through discussion with small focus groups of first and final‐year students. The first‐year focus group were six members of a bioscience tutorial group (students are allocated to these randomly) and took place in a timetabled tutorial session to encourage participation, while the final‐year group comprised seven student peers undertaking either Biochemistry or Natural Sciences degrees. Both focus group meetings were carried out via Microsoft Teams and the conversation recorded so the moderator (JB) could focus on the group's understanding of the questions posed.

The focus group opinions were sought to define the skills for university life and the workplace to be included in the initial questionnaire, and also to help inform the questionnaire design (the number and type of questions) and what type of incentive would be the most enticing. The final‐year focus ran first, giving some ideas for the questionnaire format and incentivisation, which were validated by the first‐year group (File S2). Due to the limited scope of the focus groups, no formal qualitative analysis was conducted.

### Surveying a broader range of opinions through questionnaires

Following the opinions of the focus groups, the initial anonymous questionnaire (File S3) was designed that asked for a snapshot of students' confidence in each of the 16 skills identified before they started university and after their first year (or during if they were first‐year students). It also asked about their satisfaction with the support received from the university in developing these skills. A Likert scale was used to increase the probability of return. Pre curriculum redesign, there was a formal skills module only in bioscience year 1, so the questions focussed on the first‐year experience and its value as perceived as students progressed through their courses.

After analysis of these initial data, a follow‐up questionnaire with interview‐style questions (File S4) was sent to those who expressed interest in receiving this in their response to the first. The longer‐answer questions were reserved for the follow‐up questionnaire with the aim of reducing the effort required to complete the first and hence increase participation.

### Participants

The questionnaires were sent to all undergraduate students at the University of Bath studying Biochemistry, Biomedical Sciences and Biology, who undertook a compulsory first‐year key‐skills module, and also to the Natural Sciences students, who did not. A hypothesis was that including a group of students undertaking a science degree that lacked a skills module would enable the importance of such a module to be determined. Natural Sciences is an interdisciplinary degree and distribution of the questionnaire was not restricted only to those studying Biological Sciences. This work was carried out in the autumn of 2020, thus analyses opinions during the Covid pandemic; however, for the established students, it reflected their pre‐pandemic experiences.

### Data analysis

Statistical analysis of the data was carried out using SPSS. Sample sizes (see results) were large enough to overcome the violation of the normal distribution by ordinal Likert scale data, thus paired *t*‐tests were carried out to determine if there was a significant difference between students' confidence levels in each of the 16 different skills, before university and after their first year, for each year group and degree subject. This was to analyse the hypotheses that all 16 skills should be taught at university and that a taught skills module was necessary for students to achieve these skills.

Subsequently, one‐way ANOVA and *post hoc* Tukey statistical tests were carried out on the skills that did not show a clear trend across all categories to determine if there really was a significant difference in the results between the different year groups and degree subjects. A Tukey test is run after analysis such as ANOVA (‘*post hoc*’) to compare all possible pairs of means and interpret the statistical significance of the difference between means. The method is conservative when there are unequal sample sizes [22].

Additionally, because such a large number of *t*‐tests were carried out there was a greater risk of type I error (rejection of a true null hypothesis, false positive), as there was a greater probability of chance having an influence on the results. Therefore, the Bonferroni adjustment was used to correct ‘experiment‐wise’ error rates [23]. This suggested that the new p‐value for each individual test was equal to the original *P*‐value (≤ 0.05), divided by the number of tests performed (16, one for each skill) [24]. As fewer subsequent ANOVA and *post hoc* tests were carried out, a *P*‐value of ≤ 0.05 was used for these tests. Therefore, the p‐values used were ≤ 0.003125 for the *t*‐tests and ≤ 0.05 for the one‐way ANOVA and *post hoc* Tukey tests.

To understand the effect size, Cohen's d values were calculated by dividing each *t*‐test value by the square root of the participant number in that group. The effect magnitude is interpreted as small if Cohen's d is between 0.2 and 0.5, medium if between 0.5 and 0.8 and large if greater than 0.8 [25].

## Results and Discussion

This study aimed to investigate skills development amongst first‐year biosciences and Natural Sciences students at the University of Bath, from the students' viewpoint. It was carried out in the autumn of 2020, comparing first‐year students who gave an on‐arrival opinion, with second years who had experienced first‐year skills training in a pre‐Covid environment, and final‐year students, many of whom had undertaken industrial placements and all of whom were applying for jobs or further education and could comment on the suitability of skills training. The results from focus groups defined the parameters of the study; subsequently, questionnaires were used to seek opinions from the whole cohort, the data from which were interpreted to evaluate the scope and effectiveness of the first‐year skills teaching provision and provide suggestions for improvements to the skills provision during a transformation of the university curriculum.

### The parameters of the study

The focus group activities helped to define the aims of this study, to evaluate the perception of student competencies in 16 different skills (presentation, academic writing, scientific writing, teamwork, laboratory, research, study, analytical, leadership, problem solving, organisational, adaptability, resilience, critical thinking, time management and interpersonal) and secondly to look at the role of the taught first‐year module in developing those skills.

The initial questionnaire elucidated 204 responses, about 23% of the total population. This included 64 Biochemistry students, 49 Biologists, 35 Biomedical Scientists, and 56 Natural Scientists from the first year (70), second year (51) and final years (83). The follow‐up questionnaire was answered by 14 Biochemists, 4 Biologists, 8 Biomedical Scientists, and 3 Natural Scientists, 7 first years, 4 second years and 18 final‐year students.

### Transferrable skills are valued more highly than laboratory, subject‐specific, skills

**Fig. 1:**
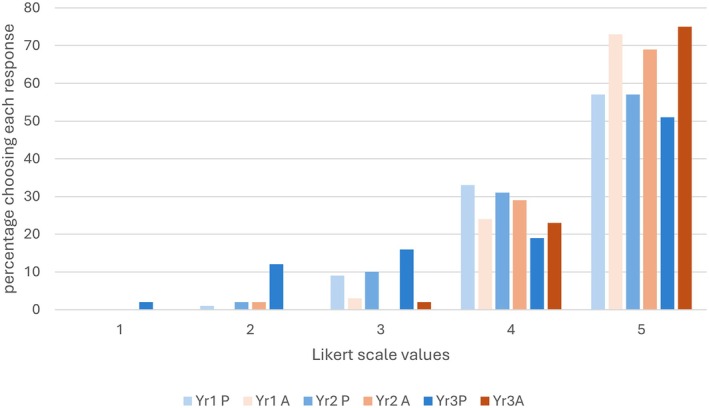
Percentage frequency distribution showing how much the students valued improving their Practical (Yr1P, Yr2P, Yr3P) skills and their Academic or Transferrable Skills (Yr1A, Yr2A, Yr3A) across the different year groups. These are the responses of the different year groups to questions 3 and 4 of the first questionnaire. The relative percentage frequencies for each point on the Likert scale are shown across the different year groups (1 = Not at all, 5 = Very much). The values for practical skills and academic/transferable skills are shown in blue and red, respectively. The sample sizes (*n*) for each category are year 1 *n* = 70, year 2, *n* = 51 and year 3 *n* = 83.

The first questions asked the students how much they valued skills development in general. Out of the 204 respondents, 73% of students valued improving their academic or transferable skills ‘very much’ (5 on Likert scale) whilst only 54% of students valued improving their practical or laboratory skills at this level. Across all years, the students valued improving their academic or transferable skills more than their practical skills, this difference increasing with the year of study (Fig. 1).

The decreasing interest in developing practical skills might be expected as most fundamental laboratory skills are mastered in year 1, with these being used for more advanced experiments in later years, when a smaller number of more specialised skills are taught. At the same time, as students mature, the aspirations of many move away from a research or laboratory career and for that cohort the practical skills will not be necessary for success. These conclusions were echoed in the follow‐up questionnaire findings for example

‘First year students like myself are too busy figuring everything else out! it (transferrable skills) doesn't feel like a big priority, when jobs that require those skills feel like ages away in the future, and my coursework deadline is next week ya know.’ First year Biochemistry student

‘When coming into university, I was mostly focussed on getting the work done to perform well in exams and get good grades. However as a final‐year student now, I have greater appreciation for the importance of transferable skills, especially looking at career prospects.’ Final Year Biology Student

and align to similar findings elsewhere [17]. Due to the ever‐increasing competition for employment between graduates [7], students are becoming more aware of employer expectations beyond their formal qualifications [8], including transferable skills such as communication, problem‐solving, critical thinking, leadership and teamwork [26].

### Student perception of their skills level before entering university and following their first year of study

**Fig. 2:**
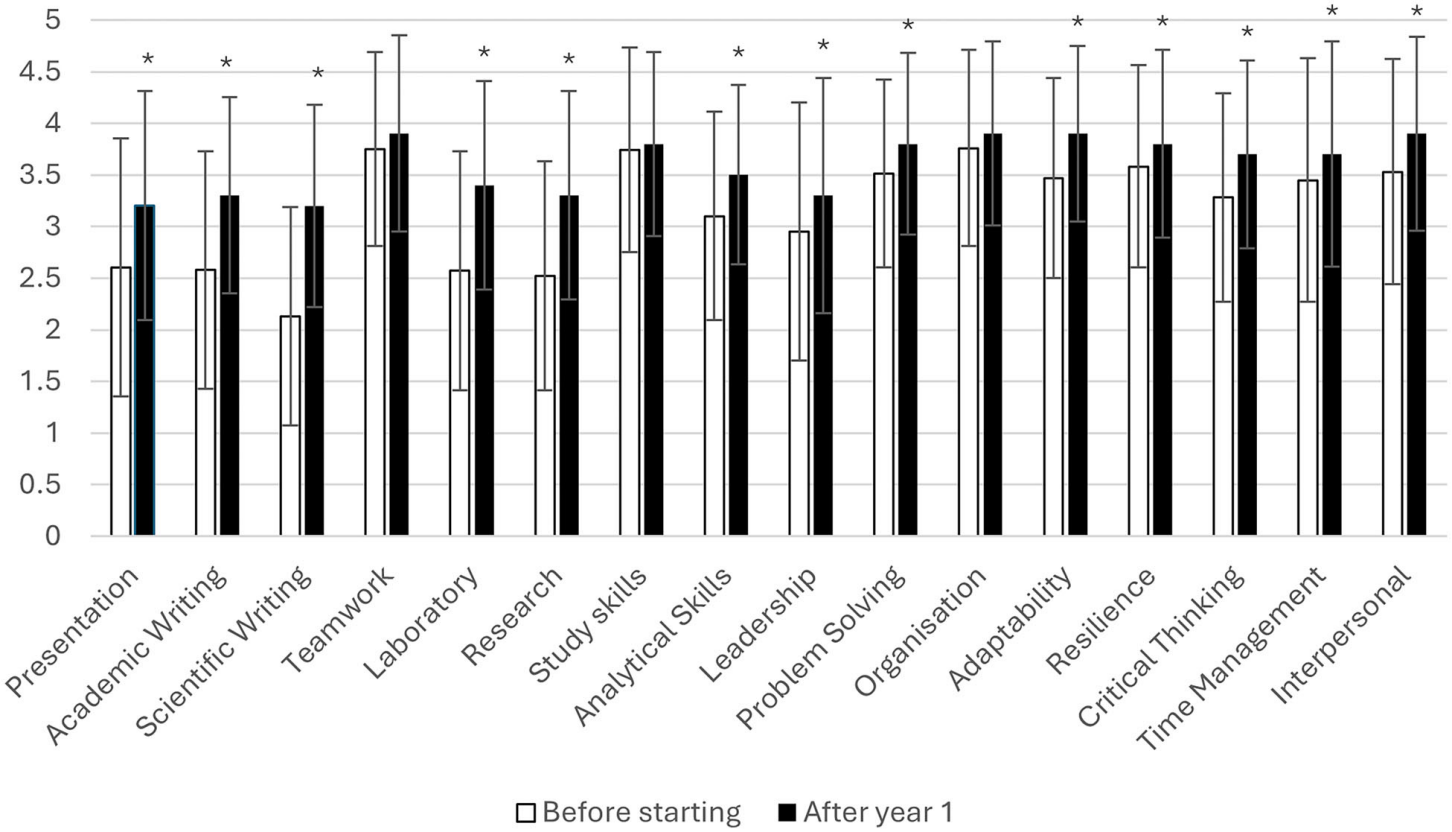
Average Likert scale values for all respondents' confidence in 16 different skills *before* starting university (white bars) and following year 1 (black bars) (*n* = 204). The Likert scale used was 1 = Not confident at all, 2 = Slightly confident, 3 = Neutral, 4 = Fairly confident, 5 = Very confident. The mean values are shown to 1 decimal place (1 d.p.) ± SD. * indicates a statistically significant paired *t*‐test *P*‐value < 0.00315 (corrected for ‘experiment‐wise’ error rates by the Bonferroni adjustment). The numerical *P*‐values are in Table 1.

The students' confidence in the 16 different skills before starting their course, and after the first year (or after the first few weeks in the case of year 1 students) were probed in the same questionnaire, with results shown in Fig. 2 and analysed in Table 1. Significantly fewer skills were proposed to have been developed in the first year by first‐year students, compared to second‐ or final‐ year students (Table 1). This is to be expected, given that first‐year students were only commenting on their experiences during the first 5 weeks at university, whereas second‐ and final‐year students were reflecting on their whole first‐year experience. Additionally, first‐year students proposed to have been ‘fairly confident’ in a greater number of skills than second‐ or final‐year students prior to starting university (Supplementary Table S1). It is likely that with experience students can reflect more accurately on their level of skills before university, through recognising how much their ability actually improved at university. The inexperience of first years is highlighted in the perception of their adaptability, which they were confident had improved, however by the second year this confidence had dissipated (Table 1). However, the significant improvement reported by first‐year students during the first weeks of university in the more technical scientific skills, such as laboratory, research and scientific writing, was also borne out by the higher years. This indicates that the university curriculum effectively introduces these technical skills at the start of the degree course. The National Research Council [27] argues that developing these scientific skills not only builds science expertise, but they may also help to improve other essential 21st century skills, such as problem solving [28].

**Table 1:** Paired *t*‐test results, with Cohen's d effect size and magnitude for each paired t‐test, for perceived improvement in 16 different skills amongst first‐year, second‐year and final‐year students during their first year at university, followed by post hoc Tukey tests to test for significance between the results where a one‐way ANOVA indicated significance. The overall results for all respondents are also shown. A significance level of ≤ 0.003125 was used for the *t*‐tests, due to the Bonferroni adjustment. A *P*‐value of ≤ 0.003125 indicated a statistically significant improvement (null hypothesis rejected). Values reported to 4 significant figures to match the significance level. Cohen's d effect size magnitudes are small (S) if between 0.2 and 0.5, Medium (M) if between 0.5 and 0.8 and Large (L) if greater than 0.8 [25]. If a one‐way ANOVA test showed a significant difference between the results obtained across the different years, post hoc Tukey tests were carried out to determine where the differences lay. A significance level of ≤ 0.05 was used, hence values are reported to 2 decimal places. Grey shading indicates no statistically significant improvement.

	Year group						Overall all respondents (*n* = 204)		Post‐hoc Tukey test	
	First‐year (*n* = 70)	Effect size magnitude	Second‐year (*n* = 51)	Effect size magnitude	Final‐year (*n* = 83)	Effect size magnitude		Effect size magnitude	1–2	1–3
Presentation	0.1093	0.19	2.131 × 10^−6^	0.75 m	6.881 × 10^−11^	0.82 L	1.725 × 10^−15^	0.60 m	0.00	0.00
Academic Writing	0.002021	0.38 S	3.731 × 10^−6^	0.73 m	6.821 × 10^−13^	0.93 L	3.645 × 10^−19^	0.69 m		
Scientific Writing	5.363 × 10^−7^	0.66 M	1.215 × 10^−11^	1.22 L	5.943 × 10^−20^	1.33 L	5.436 × 10^−33^	1.01 L		
Teamwork	0.2542	0.14	0.04710	0.29 S	0.003816	0.33 S	0.007021	0.19		
Laboratory	1.514 × 10^−6^	0.63 M	1.014 × 10^−6^	0.78 m	4.299 × 10^−13^	0.95 L	3.627 × 10^−23^	0.79 m		
Research	7.576 × 10^−6^	0.58 M	7.941 × 10^−5^	0.60 m	3.510 × 10^−15^	1.06 L	2.102 × 10^−22^	0.77 m		
Study	0.4835	0.08	0.2451	0.16	0.06163	0.21 S	0.09544	0.12		
Analytical	0.03381	0.26 S	0.004440	0.42 S	1.097 × 10^−9^	0.76 m	4.855 × 10^−11^	0.49 S	0.44	0.00
Leadership	0.007031	0.33 S	0.009803	0.38 S	1.977 × 10^−8^	0.68 m	2.532 × 10^−11^	0.49 S	0.97	0.05
Problem Solving	0.1464	0.18	0.2538	0.16	6.548 × 10^−7^	0.59 m	1.175 × 10^−5^	0.31 S	0.99	0.08
Organisational	0.08638	0.21 S	0.6726	0.06	0.02680	0.25 S	0.006671	0.19		
Adaptability	1.230 × 10^−5^	0.56 M	0.1030	0.23 S	4.4561 × 10^−9^	0.72 m	3.573 × 10^−13^	0.54 m	0.20	0.20
Resilience	0.1291	0.18	0.04848	0.28 S	0.0008233	0.38 S	3.039 × 10^−5^	0.30 S		
Critical Thinking	0.02223	0.28 S	2.519 × 10^−5^	0.65 m	1.611 × 10^−8^	0.69 m	1.030 × 10^−12^	0.53 m	0.03	0.01
Time Management	0.02970	0.27 S	0.4976	0.10	0.01891	0.26 S	0.001554	0.22 S		
Interpersonal	0.08584	0.21 S	6.423 × 10^−5^	0.61 m	1.365 × 10^−5^	0.51 m	1.234 × 10^−9^	0.45 S	0.02	0.09

### Some skills are already well‐developed pre‐arrival at university

Overall, the student body considered themselves to be ‘fairly confident’ in 8 out of the 16 skills before starting university, with teamwork, study and organisational skills having the highest average values (3.8, 3.7 and 3.8 respectively) (Fig. 2). These skills were also the only ones ranked ‘fairly confident’ across the individual year groups and subject cohorts (Supplementary Table S1), and the skills which did not show improvement in confidence during their time at university (Table 1). Improvement in perceived time management skills was also not detected within the individual cohorts, although significance and a small effect size were reached when the whole body of data was considered (Table 1), suggesting that this does improve marginally at university.

In the focus groups, time management and organisation were highlighted as being some of the most important skills for success at university:

‘… time management is one of the biggest things, ‘cause things can easily start to pile up ‘cause you're not [on] schedule of you're doing this lecture at this time, if it's a pre‐recorded one.’ First‐year Biochemistry student

‘… getting your laundry done and everything is like new for me… at home like my parents did it… now, you have to do it all on your own.’ First‐year Biochemistry student

Some students also highlighted the considerable difference between the organisation skills required during sixth form, compared to first year:

… [I] was quite organised at school, but it's really different at university, especially when there is a lot more responsibility and it's very like independent learning.’ Final‐year Natural Sciences student

Effective time management has been shown to have a positive correlation with student success, in regard to distance learning [29]. It is likely that these skills were necessary for the students to develop in order to obtain a place at university, and any subsequent activities have not required the students to hone them further, beyond the novel responsibility of living on their own which could cause additional stress and disruptions [20].

### Some skills are acquired through formal training

As shown in Fig. 2 and Table 1, students' confidence was statistically significantly improved in the remaining 12 of the 16 skills during their first year at university.

In the first few weeks of their university career the first‐year students perceived an improvement in their academic writing, scientific writing, laboratory and research skills (Table 1) which are the skills taught explicitly in the skills unit from the start of the year. Moving into the second and third year there is increasing recognition of the improvement during year 1; the perceived improvement in all these skills has large effect sizes by the final year. The influence of the explicit training in academic skills is shown by the reduced confidence in presentation skills. Training in presentation skills takes place later in the first year than the questionnaires were completed, so confidence being increased only from the second year suggests that this training is required to increase confidence in communication skills.

In the follow‐up questionnaire, students explained that they improved their scientific and academic writing skills through writing lab reports and essays. Some alluded to the fact that these skills were developed effectively using feedback that was given on these assignments:

‘I was asked to do [an] essay for my personal tutor… this helped me understand how university work is assessed compared to A‐Levels.’ Final‐year Natural Sciences student

‘I've had to learn the specific tense and voice (e.g. past passive) to use and how to ensure references have been used correctly.’ First‐year Biochemistry student

‘Receiving feedback and then being set another writing task is a great way to improve.’ Final‐year Biomedical Sciences student

In the responses to the follow‐up questionnaire students from both cohorts acknowledged that their first‐year skills training aided understanding of scientific writing, with the bioscience students acknowledging the value of the skills training module while the training was less focused but still present for Natural Scientists.

‘During my first year, my course had a specific module dedicated to scientific writing where we have weekly lectures covering different aspects of a report, how to tackle them & what is expected. Additionally, I had weekly labs where I needed to produce scientific reports.’ Final‐year Biomedical Sciences

‘The biochemistry course had a series of introduction lectures providing information on how to write each part of the report ‐ this was invaluable for improving understanding.’ Final‐year Biochemistry

‘Writing essays for my course assessment by following the structures and aims provided.’ First‐year Natural Sciences

‘Having to do essays and critically analyse studies helped get a feel for how to write in the proper way.’ Second‐year Natural Sciences

‘More consistent laboratory sessions and the lab write ups that are required with them mean we get practice at reviewing our experiments on a regular basis. I got practice in writing essays through some of my modules which was very useful in terms of learning how to structure longer pieces of work and reference.’ Final‐year Natural Sciences

Other studies have found that repeated feedback by university tutors in the first semester of first year allowed students to develop their academic writing skills promptly [19]. Feedback on student performance is viewed as one of the most powerful and effective influences on learning and achievement [30]. However, as elucidated by Shafi *et al*. [31], students use this feedback in a variety of ways, although mainly by feeding forward to the next assignment. Therefore, a focus on first‐year skills training is appropriate, focussing feedback provision earlier, as students are less likely to improve and progress from feedback given on final tasks [32].

### Skills acquired by students by the final year through means other than formal skills training

The remaining skills identified by the focus groups, (analytical, leadership, problem solving, adaptability, resilience, critical thinking, and interpersonal skills) are not explicitly addressed in formal skills teaching received by the students. Nevertheless, the respondents to the first questionnaire considered that critical thinking and interpersonal skills are significantly improved by the end of the first year at university. Adaptability had the unusual profile (Table 1) of being perceived as significantly improved during the first weeks of the first year, but then dropping back in the second year, although this change did not reach statistical significance. These skills, like the teamwork, study and organisational skills discussed earlier, are required to be developed if the students are to succeed in university study, but unlike those already discussed, may not be required until the student reaches university, hence the perception that they have developed later. These findings correspond with those of Holman [33] and Lucas et al. [34], who propose that students view skill development as a tacit process. Lucas et al. concluded that students consider skills to develop naturally from personality types or as one grows older. In this study, students recognised that their course of higher education aided their skills development, albeit indirectly, rather than directly.

By the final year, confidence in analytical, leadership, problem solving, and resilience skills was also significantly improved over that reported in years 1 or 2. These are not part of the skills training, but may have been enhanced through the opportunity to go on placement, most obviously resilience:

‘… so frequently, experiments don't go well and that just happens in the real world… being able to systematically identify the cause of failure in the real world is a real skill I've got outside of university.’ Final‐year Biochemistry student (lab‐based placement)

‘…I don't think anyone ever tells you how many rejections you're going to get.’ Final‐year Biochemistry student (non‐lab‐based placement), speaking about job applications.

Respondents of the follow‐up interview questionnaire suggested ways in which training in resilience could be incorporated into the first‐year curriculum, which included resubmitting the same assignments again following effective feedback from tutors, participation in real‐life investigations and talks from professional guest speakers. The following are some example responses:

‘… maybe if we got a chance to improve it after getting comments back on it, we would learn to keep going at things until we get them right?’ First‐year Biochemistry student.

‘… students could contribute more to professors’ investigations, even with simpler wet‐lab projects… way more value than conducting a dull experiment to achieve an ordinary result.’ Final‐year Biochemistry student

‘… hearing experiences early on… professionals will teach us that failure is ok.’ Final‐year Natural Sciences student

Resilience is especially important in the practice of problem solving, as the ability of a person to overcome their problems is an indicator of their resilience [35]. As well as creativity, imagination, insight and success; disappointment, difficulty and failure are also common stages of an explanation building process [36]. However, starting a class with a problem may require more time than if the content was taught directly, via a teacher‐centred approach, therefore this may not always be possible given the time constraints of an undergraduate degree [37].

Respondents of the follow‐up questionnaire proposed that training in problem solving could also be incorporated by students designing laboratory experiments and also through frequent problem‐solving worksheets, workshops and quizzes every few weeks. Example comments included:

‘If labs were based more around solving a problem (i.e. designing a synthesis to perform) rather than following a set of instructions.’ Second‐year Natural Sciences student

‘Little problem‐solving quizzes involved every couple of weeks just to practice not to be graded but should be compulsory.’ First‐year Natural Sciences student

‘Have fun tasks and workshops where students use their problem‐solving skills to solve a scenario.’ Final‐year Natural Sciences student

‘Regular formative problem sheets with your tutor and discuss afterwards, as a group, the solution.’ Final‐year Biochemistry student

The majority of respondents to the follow‐up questionnaire suggested group work as a method of improving leadership skills amongst first‐year students, as well as debate/discussion sessions. Examples included:

‘I think the best way to test leadership skills is to set group tasks. This is the best way for each individual in the group to have moments of leadership and it also builds teamwork skills at the same time. After the task is completed, the evaluation could focus not just on the content but the process of working as a group and as a leader to achieve said task.’ First‐year Biochemistry

‘Group projects: work in a team in practicals then design and deliver a presentation together.’ Final‐year Natural Sciences

‘Assigning groups and then keeping the groups the same but changing who leads a project or task.’ Final‐year Natural Sciences

‘Assign lab partners randomly and assign groups randomly. This puts students outside their comfort zone, requiring them to be more active and push themselves more.’ Final‐year Biochemistry

**Fig. 3:**
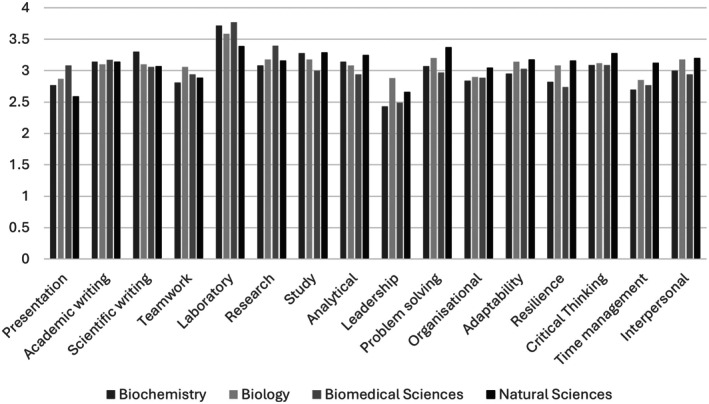
Student satisfaction with the support received from the University of Bath in aiding the development of 16 different skills during the first year of university. These are the average Likert scale responses for all 16 skills (Presentation, Academic Writing, Scientific Writing, Teamwork, Laboratory, Research, Study, Analytical, Leadership, Problem Solving, Organisational, Adaptability, Resilience, Critical Thinking, Time Management and Interpersonal), the responses to question 7 of the first questionnaire for each of the cohorts (Biochemistry *n* = 64, Biology *n* = 49, Biomedical Sciences *n* = 35, Natural Sciences *n* = 56).

These groupwork suggestions align well with existing research, which suggests that leadership is a collective phenomenon that is shared amongst people [38]. Leadership was the skill in which the students were least satisfied with the support they had received, (Fig. 3), so incorporating some of these suggestions could better support their learning. Leadership through laboratory group work has shown an improvement in perceived career skills in Chemistry [39].

### Is a designated skills module required for perception of skills acquisition?

This study included students studying bioscience degrees (who had formal skills training module) and those studying Natural Sciences (who did not) and one hypothesis of this study was that there would be a significant difference between the skills developed by biosciences students, compared to Natural Sciences students. The statistical significance of the perceived improvement in presentation, analytical, leadership, problem solving, adaptability and interpersonal skills during university study did differ between disciplines (Table 2). However, these differences were present not only between biosciences and Natural Sciences, but also amongst the different bioscience degree cohorts and were subsequently found not to be statistically significant via one‐way ANOVA tests (results not shown). This suggests that Natural Sciences students also gained these skills via other means. Similarly, the satisfaction of Natural Sciences students with the support received in developing these skills was not significantly different from that reported by bioscience students (Fig. 3). Natural Sciences students were, on average, equally as satisfied with the skills provision received from the university as Biochemistry students (Fig. 3). Together, these students were slightly less satisfied than Biology and Biomedical Sciences students. Notwithstanding, there was a slightly smaller sample size for Biology and Biomedical Science students, compared to Biochemistry and Natural Sciences, which may have influenced the results [40].

**Table 2:** Paired *t*‐test results for perceived improvement in 16 different skills amongst Biochemistry, Biology, Biomedical Sciences and Natural Sciences students (across all years) during first year at university. A significance level of ≤ 0.003125 was used, due to the Bonferroni adjustment, to reduce type I error. The null hypothesis was that there was no significant improvement in the different skills during their first year. Cohen's d effect sizes and magnitudes are shown (magnitudes were small (S) if between 0.2 and 0.5, Medium (M) if between 0.5 and 0.8 and Large (L) if greater than 0.8 [25]). Grey shading indicates no statistically significant improvement.

	Biochemistry (*n* = 64)	Effect size	Biology (*n* = 49)	Effect size	Biomedical sciences (*n* = 35)	Effect size	Natural sciences (*n* = 56)	Effect size
Presentation	3.946 × 10^−6^	0.63 m	9.881 × 10^−6^	0.71 m	5.317 × 10^−6^	0.91 L	0.005557	0.39 S
Academic Writing	4.580 × 10^−6^	0.63 m	8.265 × 10^−5^	0.61 m	1.991 × 10^−6^	0.97 L	4.620 × 10^−6^	0.68 m
Scientific Writing	5.018 × 10^−10^	0.92 L	9.988 × 10^−12^	1.27 L	2.885 × 10^−6^	0.95 L	9.129 × 10^−10^	0.99 L
Teamwork	0.1316	0.19	0.02172	0.34 S	0.4749	0.12	0.8108	0.03
Laboratory	7.671 × 10^−8^	0.76 m	1.666 × 10^−6^	0.78 m	5.044 × 10^−7^	1.04 L	4.111 × 10^−6^	0.68 m
Research	3.206 × 10^−7^	0.71 m	7.324 × 10^−7^	0.81 L	3.121 × 10^−7^	1.07 L	2.121 × 10^−5^	0.62 m
Study	0.005547	0.36 S	0.4895	0.10	0.5542	0.10	0.5319	0.08
Analytical	0.005695	0.36 S	0.001353	0.49 S	0.0009059	0.61 m	7.490 × 10^−5^	0.57 m
Leadership	0.009791	0.33 S	2.556 × 10^−5^	0.67 m	0.02304	0.40 S	6.413 × 10^−5^	0.58 m
Problem Solving	0.004351	0.37 S	0.0007746	0.51 m	0.2010	0.22 S	0.3578	0.12
Organisational	0.04656	0.25 S	0.4183	0.12	0.1817	0.23 S	0.2116	0.17
Adaptability	0.003172	0.38S	1.362 × 10^−5^	0.69 m	0.005258	0.50 m	4.750 × 10^−6^	0.68 m
Resilience	0.04118	0.26 S	0.06733	0.27 S	0.01206	0.45 S	0.03776	0.28 S
Critical Thinking	0.0001554	0.50 m	0.002157	0.46 S	4.412 × 10^−6^	0.92 L	0.0009768	0.47 S
Time Management	0.008737	0.34 S	0.2831	0.16	0.02994	0.38 S	0.6416	0.06
Interpersonal	0.01495	0.31 S	0.0001537	0.59 m	0.0006515	0.63 m	0.01774	0.33 S

The Natural Scientists are a less uniform cohort, studying sciences not confined to biosciences, so skills training must have been provided through classes in other disciplines, not labelled as skills modules. This was confirmed in answers to the follow‐up questionnaire asking where they improved their academic writing:

‘During labs writing reports‐ physics labs in particular.’ Second‐year Natural Sciences

‘I wrote many laboratory reports after conducting practical experiments. Also, I have done presentations, and literature reviews.’ Final‐year Natural Sciences

‘I was asked to do a essay for my personal tutor ‐ he marked it critically in my first term of 1st year, this helped me understand how university work is assessed compared to A‐Levels.’ Final‐year Natural Sciences

Thus, the Natural Scientists were taught academic and practical skills during the first year as part of other activities, so proved not to be an appropriate comparator in judging the effect of formal skills training on bioscience students.

Scott [16] found that students were much more aware of the skills that they developed through explicit training than those developed implicitly through their degree course. Other methods of making skills acquisition apparent such as the use of transferrable skills badges are also successful [41]. In our study, this is indicated in the responses to the question on satisfaction with skills training in the first questionnaire (Fig. 3). The students are most satisfied with their training in laboratory skills, the skill that is most explicitly taught to them in a unique environment. Thus, our data do not demonstrate a clear advantage of a standalone skills module over embedded education, provided that the skills teaching is incorporated into other activities carried out by the students.

### Limitations of the methods

As with many such studies, these findings are subject to several limitations. Firstly, there could have been misinterpretations of the questions, both in the focus group discussions and in the questionnaires, which could have led to inaccurate responses. This was reduced as much as possible by keeping questions clear and concise and not asking two questions in one, which could be misleading [42]. Additionally, respondents may have provided dishonest answers or ones believed to be socially acceptable, particularly during focus group discussions [43]. To decrease the likelihood of this, they were assured of their confidentiality prior to expressing their views and opinions. A further limitation of the focus group discussions could have been the likelihood for certain individuals to dominate the discussion process [42]. This possibility was also reduced through the moderator stepping in when necessary to ensure that all participants contributed to the discussion.

The use of a 5‐point Likert scale, with a ‘Neutral’ option, in the initial questionnaire was another potential limitation. Previous studies have proposed that respondents may incorrectly select the midpoint, as it has been found to represent ‘undecided’ as well as ‘neutral,’ which is its true meaning [44]. Nevertheless, omitting the midpoint would have removed the option for respondents to express their neutral opinion and could have also led to biased opinions by forcing respondents to choose a side [45].

Additionally, as this study relied on student opinions, which are prone to bias, the extent of students' actual improvement in the various skills could not be established definitively.

Finally, this was a cross‐sectional study, questioning all years at one point in time, rather than following the cohorts of students with time. Thus, the study relied on the students' retrospective recall of their skill level in each skill. For the first years, recalling only 5 weeks, the recollection would be reliable, but final‐year students who had been on placement would be recalling levels from 3 years previously; the accuracy of which is a limitation of this study.

### Future directions

This study took place amongst a particular snapshot of students, mostly focussed on those studying bioscience degrees, at a particular point in time. This could be expanded in many ways. Firstly, this is mostly a pre‐COVID study; another similar poll carried out now could examine the effect of COVID restrictions on development of skills pre‐university and whether the training needs have changed. Other skills identified in the literature since our study [46] could also be evaluated for student opinions in focus groups and, if appropriate, included in a future poll.

The poll could be carried out later in the academic year to give first‐year students a chance to reflect on their whole academic year and therefore yield more considered results. However, due to the focus on examinations, participation might be considerably lowered later in the year and it is the higher return that has rendered this study more significant.

An alternative might be a longitudinal study where a cohort is defined and followed through their university career to allow pairwise comparison of perceptions in the first and final year, although the ethical implications of this would be greater as students would have to be identified, at least while the study was in progress, and student's acceptance and consent might be withdrawn. A more straightforward improvement would be to separate those who have done a placement who have expanded opportunities for skills development from those who haven't, although the separation would reduce the cohort size in the final year.

This optimised protocol could then be expanded to other biosciences departments to examine the wider applicability of these results, and discussions towards this are underway.

Moving away from relying on perceptions, future studies could incorporate a process through which proficiency is measured in the various skills before and after first year, for example, a skills test. This would also give students a clear idea of their skills level and encourage them to work harder to improve this. However, this should be voluntary, not a formal process to avoid discouragement in the first weeks as they adapt to university life.

## Conclusions

This study's findings agreed with previous work in showing that students valued both practical and transferrable skills acquisition, and also in the perceived importance of transferrable skills increasing with progression through their studies as students become more focussed on securing graduate opportunities, perhaps outside science.

The overall findings from the initial questionnaire (*n* = 204) indicated that in four of the skills: teamwork, study, organisation and time management, there was little or no significant perceived improvement during the first year at university due to the students being already quite confident with these skills prior to embarking on their degree courses. These are unlikely to require targeted activities within a transformed skills module. Similarly, for adaptability, critical thinking, and interpersonal skills which are developed as a student moves through their university career.

The academic skills (academic writing, scientific writing, laboratory and research skills) are developed by students during their first year whether within a formal module (bioscience students) or on an ad hoc basis through assignments (Natural Sciences students). For those receiving it, formal methods of skills training within a module were recognised as effective in teaching academic and practical skills. For the others, these skills were obtained from effective feedback on assignments in other modules. Academic and scientific writing skills and laboratory and research skills require teaching from the start of the first year, as is already widely embedded across degree courses.

Statistical significance for improvement in analytical, leadership, problem solving, adaptability, resilience, critical thinking and interpersonal skills varied amongst year groups and subjects (Tables 1 and 2). This would suggest that these skills could be targeted in formal teaching. Suggestions of ways that leadership, resilience and problem‐solving skills could be incorporated into teaching were received in answers to the follow‐up questionnaire and could be used to improve the first‐year skills provision within the biosciences courses during the transformation of the university curriculum.

Addressing the hypotheses that underpinned this work, not every skill identified requires formal teaching: four of the 16 (teamwork, study, organisation and time management) skills are effective before arrival and students are satisfied with gathering three more (critical thinking, adaptability and interpersonal skills) inherently through being at university. Analytical, problem‐solving, resilience and leadership skills are improved by the final year, although it was thought these could be gained more readily through formal training, alongside the remaining skills already included in the skills module.

The second hypothesis, that gathering the teaching into a formal skills module is required to recognise that the skills have been gained, is more difficult to judge as the Natural Sciences cohort may not have been an appropriate comparator group as we learnt they also receive training in laboratory skills and some academic skills in other modules. However, all students were most satisfied with their improvement in laboratory skills, the only one all are taught formally, hinting that formal training helps recognition of the skills acquired.

Overall, students were only ‘fairly satisfied’ with how the university aids their development of these skills, leaving plenty of room for improvement, particularly targeting those with which they were least satisfied such as leadership. Future studies should evaluate these implemented changes both within the university and in partnership with other institutions.

## Conflict of interest

The authors declare no conflicts of interest.

## Author contributions

JB conceived the project in consultations with SJC, carried out the focus group interviews, devised both questionnaires and did the statistical analysis in collaboration with the statistics advisory service at the university of Bath. SJC applied for ethical approval, discussed the project at all stages with JB, emailed out the initial questionnaire and wrote the paper based on JB's report submitted as part of her BSc requirements.

## Supporting information


**Table S1.** Skills that students were ‘fairly confident’ in *before* starting university for each individual category (year group and subject).
**Fig. S1.** Focus group information sheet and consent form.
**Fig. S2.** Discussion prompts used in the focus groups.
**Fig. S3.** Initial Study questionnaire.
**Fig. S4.** Skills at university—Follow‐up Interview Questionnaire.

## Data Availability

The data are analysed in this paper; raw anonymous data can be obtained on request.
