# Supplementary material for: The skills required for transition to university and study in biological sciences: A student perspective
Source: FEBS Open Bio. 2026 Mar 31;16(8):1615–29. doi: 10.1002/2211-5463.70234 (PMC13398817; doi:10.1002/2211-5463.70234)
Supplement: Supplementary file 1 — Table S1. Skills that students were ‘fairly confident’ in before starting university for each individual category (year group and subject). Fig. S1. Focus group information sheet and consent form. Fig. S2. Discussion prompts used in the focus groups. Fig. S3. Initial Study questionnaire. Fig. S4. Skills at university—Follow‐up Interview Questionnaire. [file FEB4-16-1615-s001.docx]

**Supplementary Table 1: Skills that students were ‘fairly confident’ in *before* starting university for each individual category (year group and subject)**. Average mean confidence values are shown to 1 decimal place (1 d.p.) ± SD for those where the average was greater than 3.5, i.e. the point at which where the average confidence level were closer to ‘fairly confident’ (4) than neutral (3). The three skills that students were ‘fairly confident’ in across all categories were teamwork, study and organisational. These are highlighted in the table (grey).

| **Student category** | **Skills** | **Mean confidence value (1 d.p.) ± SD** |
| --- | --- | --- |
| **First-year** | Teamwork | 3.9 ± 0.9 |
|  | Study | 3.9 ± 0.8 |
|  | Problem Solving | 3.7 ± 0.8 |
|  | Organisational | 3.9 ± 0.9 |
|  | Adaptability | 3.6 ± 0.9 |
|  | Resilience | 3.8 ± 0.8 |
|  | Critical Thinking | 3.7 ± 0.8 |
|  | Time Management | 3.6 ± 1.1 |
|  | Interpersonal | 3.7 ± 1.0 |
| **Second-year** | Teamwork | 3.7 ± 1.1 |
|  | Study | 3.6 ± 1.2 |
|  | Problem Solving | 3.5 ± 1.1 |
|  | Organisational | 3.7 ± 1.0 |
|  | Adaptability | 3.7 ± 1.0 |
|  | Resilience | 3.7 ± 0.1 |
|  | Interpersonal | 3.5 ± 1.3 |
| **Final-year** | Teamwork | 3.7 ± 0.8 |
|  | Study | 3.7 ± 1.0 |
|  | Organisational | 3.8 ± 0.9 |
|  | Interpersonal | 3.5 ± 1.0 |
| **Biochemistry** | Teamwork | 3.7 ± 1.0 |
|  | Study | 3.8 ± 0.9 |
|  | Organisational | 3.7 ± 1.0 |
|  | Time Management | 3.5 ± 1.1 |
|  | Interpersonal | 3.6 ± 1.1 |
| **Biology** | Teamwork | 3.6 ± 1.0 |
|  | Study | 3.6 ± 1.1 |
|  | Organisational | 3.8 ± 1.1 |
|  | Resilience | 3.8 ± 0.9 |
|  | Time Management | 3.5 ± 1.2 |
| **Biomedical Sciences** | Teamwork | 3.6 ± 1.0 |
|  | Study | 3.5 ± 1.1 |
|  | Problem Solving | 3.5 ± 0.9 |
|  | Organisational | 3.8 ± 0.7 |
|  | Adaptability | 3.5 ± 1.0 |
| **Natural Sciences** | Teamwork | 4.0 ± 0.7 |
|  | Study | 4.0 ± 0.9 |
|  | Problem Solving | 3.9 ± 0.8 |
|  | Organisational | 3.9 ± 0.9 |
|  | Adaptability | 3.6 ± 1.0 |
|  | Resilience | 3.8 ± 0.9 |
|  | Critical Thinking | 3.6 ± 0.8 |
|  | Time Management | 3.7 ± 1.2 |
|  | Interpersonal | 3.9 ± 1.0 |

Supplementary Figure 1: Focus group information sheet and consent form

I would like to invite you to participate in a focus group discussion regarding academic/transferrable and laboratory skills, for an undergraduate research project being undertaken at the University of Bath. Please take the time to read this information sheet carefully before consenting to take part. Also, feel free to ask any questions that you may have surrounding the focus group discussion or the subject matter itself.

My name is Janella Borrell and I am a final-year BSc Biochemistry student at the University of Bath. I am undertaking my final-year project, which involves looking into the acquisition of skills at university, as part of a greater strategy to transform the curriculum across the university. Your opinions and views will hopefully provide us with a better insight into student views surrounding academic/transferrable and laboratory skills.

Your confidentiality will be protected under the General Data Protection Regulation (GDPR) (EU) (2016). Therefore, your personal data will not be stored and your identities will not be disclosed to anyone outside of the research team. Any views or opinions expressed during the focus group discussion will be confidential and anonymised.

At the end of this research, the results will be shared with you, so that you can recognise how your contributions have aided the research process as a whole.

If you have any questions please contact Janella Borrell.

If you are happy to participate in the focus group study, please sign the consent form below.

__________________________________________________________________________________

Please tick the boxes below to confirm that you understand/agree with the following statements:

I have read the information sheet (above) and had the opportunity to ask questions.

I understand that my participation is voluntary and that I am free to withdraw at any time, up until a week after the focus group discussion has been conducted.

I understand that, should I not wish to answer any particular question or questions, I am free to decline.

I agree to take part in this focus group discussion.

________________________ _____________________ _____________________

Name of participant Date Signature

___________________________ _________________________ ________________________

Name of moderator Date Signature

Supplementary Figure 2. Discussion prompts used in the focus groups

1. Final year focus group (took place in week 3 of their final year)

1. What skills, in general, do you feel have aided your university life as a whole?

2. What skills did you have and what skills did you not have yet when leaving secondary school?]

3. During the first semester of university, what skills do you feel were targeted and you thought that really helped? You can give examples of any modules you did or anything that sort of targeted these specific skills that you felt you really learned during that first semester?

4. What skills, gained from university, do you feel aided you the most from your transition from university to a working atmosphere?

5. Which academic skills do you feel like you didn't have when you went from university to the workplace?

6. What would be a good incentive for you to answer a questionnaire?

7. How many questions would you be likely to answer in such a questionnaire?

2. First year focus group (Week 4 of their first year, changed slightly in view of the results of the first focus group)

1. What skills do you believe have aided your university life so far?

2. What level do you think that you were at in terms of academic skills earlier on this year when leaving secondary school, before starting university?

3. How much do you think your skills have improved in these first four weeks at university, and give examples

4. What kind of skills that you're learning now at university, do you think will help you when you move from university to the workplace?

5. How many questions would you be likely to answer in a given questionnaire?

6. If there was a small incentive, like a voucher or chocolates, how many questions would you be likely to answer on such a questionnaire?

7. Would you want to just answer multiple choice questions or would you answer some multiple choice, some written?

Supplementary Figure 3: Initial Study questionnaire sent out during the 4^th^ week of the academic year (as a Microsoft form). The GDPR statements have been omitted for clarity. All of questions 3 to 7 used a Likert scale.

1. Which course do you do? Biology/Biochemistry/Biomedical Sciences/Natural Sciences
2. Which degree year are you in? First/Second/Placement/Gap/Final(third)/Masters/other
3. How much do you value improving your practical skills?
   1. Not at all 1 2 3 4 5 Very much
4. How much do you value improving your academic/transferrable skills?
   1. Not at all 1 2 3 4 5 Very much
5. How confident were you with the following skills BEFORE starting university?

|  | Not confident at all | Slightly confident | Neutral | Fairly confident | Very confident |
| --- | --- | --- | --- | --- | --- |
| Presentation |  |  |  |  |  |
| Academic writing |  |  |  |  |  |
| Scientific writing |  |  |  |  |  |
| Teamwork |  |  |  |  |  |
| Laboratory |  |  |  |  |  |
| Research |  |  |  |  |  |
| Study |  |  |  |  |  |
| Analytical |  |  |  |  |  |
| Leadership |  |  |  |  |  |
| Problem Solving |  |  |  |  |  |
| Organisational |  |  |  |  |  |
| Adaptability |  |  |  |  |  |
| Resilience |  |  |  |  |  |
| Critical thinking |  |  |  |  |  |
| Time management |  |  |  |  |  |
| Interpersonal (communicating with other people) |  |  |  |  |  |

1. How confident were you with the following skills AFTER your first year at university (or PRESENTLY if you are a first-year student)?

|  | Not confident at all | Slightly confident | Neutral | Fairly confident | Very confident |
| --- | --- | --- | --- | --- | --- |
| Presentation |  |  |  |  |  |
| Academic writing |  |  |  |  |  |
| Scientific writing |  |  |  |  |  |
| Teamwork |  |  |  |  |  |
| Laboratory |  |  |  |  |  |
| Research |  |  |  |  |  |
| Study |  |  |  |  |  |
| Analytical |  |  |  |  |  |
| Leadership |  |  |  |  |  |
| Problem Solving |  |  |  |  |  |
| Organisational |  |  |  |  |  |
| Adaptability |  |  |  |  |  |
| Resilience |  |  |  |  |  |
| Critical thinking |  |  |  |  |  |
| Time management |  |  |  |  |  |
| Interpersonal (communicating with other people) |  |  |  |  |  |

1. How satisfied were you with the support that you received from the university during your first year in enabling you to develop the following skills?

|  | Not satisfied at all | Slightly satisfied | Neutral | Fairly satisfied | Very satisfied |
| --- | --- | --- | --- | --- | --- |
| Presentation |  |  |  |  |  |
| Academic writing |  |  |  |  |  |
| Scientific writing |  |  |  |  |  |
| Teamwork |  |  |  |  |  |
| Laboratory |  |  |  |  |  |
| Research |  |  |  |  |  |
| Study |  |  |  |  |  |
| Analytical |  |  |  |  |  |
| Leadership |  |  |  |  |  |
| Problem Solving |  |  |  |  |  |
| Organisational |  |  |  |  |  |
| Adaptability |  |  |  |  |  |
| Resilience |  |  |  |  |  |
| Critical thinking |  |  |  |  |  |
| Time management |  |  |  |  |  |
| Interpersonal (communicating with other people) |  |  |  |  |  |

Thank you

Supplementary Figure 4: Skills at university – Follow-up Interview Questionnaire

Hi again. Firstly, thank you very much for answering my previous questionnaire entitles ‘Skills at university’ and for providing your email at the end.

As you are aware, this was carried out in order to gain an insight into the perceptions of skills development among undergraduate students studying biosciences and Natural Sciences at the University of Bath. I have now analysed the response and, as a result, I have a few questions that I would like to ask you further, surrounding these topics.

Again, please note that your confidentiality will be protected under the General Data Protection Regulation (GDPR) (EU) (2016) and your identities will not be disclosed to anyone outside of the research team. Any answers received will remain confidential and will be anonymised.

Thank you very much for taking part in this follow-up interview-style questionnaire.

1. Why do you think that final-year students expressed a greater value in improving their academic/transferable skills than first- or second-year students? If you are a final-year student, please feel free to comment on your personal experiences.
2. Overall, the results showed that students significantly improved their Academic Writing and Scientific Writing skills during first year. Can you please provide examples of how you believe that you improved these skills during this time?
3. Furthermore, the results suggested that students did not significantly improve their resilience during this time. Could you please give examples of possible ways in which training in resilience could be incorporated into the first-year curriculum?
4. Additionally, do you have any ideas of ways in which training in Problem Solving skills could also be incorporated?
5. Finally, could you please give examples of how training in Leadership skills could be incorporated into the first-year curriculum?
6. The skills that were included in the previous questionnaire are as follows: Presentation, Academic Writing, Scientific Writing, Teamwork, Laboratory, Research, Study, Analytical, Leadership, Problem Solving, Organisational, Adaptability, Resilience, Critical Thinking, Time Management and Interpersonal. Aside from these, are there any other skills that you wish you would have had more help in developing during first year? If possible, please explain why.
